# Application of novel plasma separation filter cards for quantification of nucleoside/nucleotide reverse transcriptase inhibitor di/triphosphates in dried blood spots using LC–MS

**DOI:** 10.4155/bio-2023-0057

**Published:** 2023-06-09

**Authors:** Beth Thompson, Sujan Dilly-Penchala, Alieu Amara, Helen Reynolds, Saye Khoo, Laura Else

**Affiliations:** 1 Department of Pharmacology & Therapeutics, Institute of Integrative, Systems & Molecular Biology, University of Liverpool, L7 8TX, UK; 2 Bioanalytical Facility, University of Liverpool, William Henry Duncan Building, Liverpool, L7 8TX, UK; 3 Royal Liverpool University Hospital, Liverpool, L7 8XP, UK

**Keywords:** adherence, antiretrovirals, dried blood spots, emtricitabine triphosphate, lamivudine triphosphate, LC–MS, tenofovir diphosphate

## Abstract

**Background:**

A rapid and sensitive LC–MS method has been developed and validated for the quantification of nucleoside di/triphosphates using a novel plasma separation card (HemaSep).

**Materials & methods:**

Cards were spotted with whole blood and stored at -80°C. Metabolites were extracted using 70:30 MeOH:20% formic acid, followed by weak anion exchange SPE and eluted using a Biobasic-AX column. Quantification was performed using a triple quadrupole mass spectrometer with a calibration range of 1.25–250 pmol/sample.

**Results:**

The recovery of metabolites was high (>93%). Precision and accuracy were acceptable and metabolites remained stable on the card after 29 days (stored at ambient temperature).

**Conclusion:**

HemaSep dried blood spots are a useful microsampling tool and offer an alternative to liquid plasma as they maintain stability over time.

The nucleoside/nucleotide reverse transcriptase inhibitors (NRTIs) form the backbone of most first- and second-line antiretroviral therapy (ART) regimens prescribed to treat human immunodeficiency virus (HIV)-1 infection. They are also administered as pre-exposure prophylaxis (PrEP) treatment for those at high risk of infection [1]. NRTI are prodrugs that require phosphorylation by intracellular nucleoside kinases to their active triphosphate form (NRTI-TP).

Tenofovir (TFV) is co-formulated with emtricitabine (FTC) as the prodrug tenofovir disoproxil fumarate (TDF), or more recently as tenofovir alafenamide (TAF). TAF is administered at a lower dose and is associated with reduced parent TFV plasma exposures as compared with TDF while achieving up to sevenfold higher concentrations of the intracellular active phosphorylated moiety (tenofovir diphosphate [TFV-DP]) in HIV target cells [2].

Measurement of NRTI concentrations in plasma is standard practice for the monitoring of adherence to ART, and for the evaluation of pharmacokinetics (PK) in specific populations. Plasma remains the ‘gold standard’ matrix for antiretroviral drug quantification; however, in order to obtain plasma for such purposes, specialized laboratory facilities and trained staff are required. This includes transport and storage at low temperatures to maintain sample stability, which can be costly and inaccessible in resource-limited settings. The benefits of dried blood spots (DBSs) as an alternative sampling strategy in PK trials include simplified collection, reduced invasiveness of the procedure, and minimal processing and storage requirements. DBSs can be shipped at ambient temperature and pose a reduced infection risk to laboratory personnel upon drying [3].

Due to their short elimination half-lives, direct measurement of parent NRTI in plasma can only provide a measure of recent or ‘white coat’ adherence [4]. TFV, FTC and lamivudine (3TC) have respective plasma half-lives of 14 h, 10 h and 3–4 h [5,6]. By contrast, the active NRTI-TP are known to persist in red blood cells (RBCs) and/or peripheral blood mononuclear cells (PBMCs) for longer [7], and thus provide a more reliable measure of a patient's long-term or cumulative adherence. TFV-DP, in particular, has been shown with repeated dosing to accumulate in RBC with a half-life of 17 days [8,9]. The metabolite of FTC, emtricitabine triphosphate (FTC-TP), has been found to have a half-life of 1.5 days in RBC [10]. Currently there is no data detailing the half-life of lamivudine triphosphate (3TC-TP) in RBC; however, Durand-Gasselin *et al.* found intracellular levels of 3TC-TP in RBC to be between 0.04 and 0.13 pmol/10^6^ cells [11].

A number of analytical methods for quantification of intracellular NRTI-TP in DBS have been developed and utilized for NRTI adherence monitoring [8,9,12,13]. However, many are complex and labour-intensive involving ‘indirect’ quantification of the NRTI-TP, by converting the phosphorylated form back to the parent drug via enzymatic digestion by phosphatases and multiple SPE steps [8,12]. Moreover, the majority of DBSs are traditionally collected and processed whole blood on Whatman 903 Protein Saver cards [2,4,7–10,12–16]. A limitation of Whatman DBS is that all components of the whole blood are contained within the spot, which does not allow for determining the distribution of the NRTI-TP within separate cellular and plasma fractions of the blood. Traditional DBSs are also sensitive to variations in hematocrit [17], and drug concentrations are not always correlated with plasma due to drug partitioning into erythrocytes [18].

Plasma separation cards are a novel microsampling technology that have recently become commercially available and may help to overcome some of the limitations of DBS. The HemaSep™ (Ahlstrom-Munskjö, Helsinki, Finland) plasma separation filter card uses lateral flow technology to rapidly separate upon contact the plasma from the cellular fraction in whole blood without need for centrifugation. The samples dry in a matter of hours and can then be shipped at ambient temperature for quantitative analysis. The instantaneous separation of plasma and cellular fractions from whole blood would allow for simultaneous detection of both parent NRTI in the plasma, as well as the intracellular metabolite within the cellular fraction.

Here we report a novel LC–MS method for direct quantification of phosphorylated metabolites TFV-DP, FTC-TP and 3TC-TP from whole blood spotted onto HemaSep plasma separation filter cards. The method was validated in accordance with US FDA guidelines using whole blood collected from people living with HIV (PLWH) receiving either TDF- or TAF-based ART [19].

## Materials & methods

### Reagents & equipment

TFV-DP, FTC-TP, 3TC-TP and ^13^C_5_-TFV-DP internal standard (IS) were purchased from Toronto Research Chemicals (Toronto, Canada). Methanol, acetonitrile, etc. were purchased from Thermo Fisher Scientific (Hemel Hempstead, UK). HemaSep™ plasma separation cards were purchased from Ahlstrom-Munksjö (Helsinki, Finland). Human whole blood was purchased from the National Health Service National Blood Bank (National Health Service Blood and Transplant, Liverpool, UK).

The LC–MS system consisted of a front-end Shimadzu Nexera^®^ X2 uHPLC system and autosampler (Milton Keynes, UK) connected to an AB SCIEX 5500 triple quadrupole mass spectrometer interfaced with an ESI source (AB Sciex UK Limited, Warrington, UK).

### Chromatographic & mass spectrometric conditions

TFV-DP, FTC-TP, 3TC-TP and ^13^C_5_-TFV-DP IS were eluted on a Thermo Fisher BioBasic™ AX 50 × 1 mm column. This particular column was selected due to its ability to retain highly polar molecules. Due to nucleoside di/triphosphates being comprised of two/three phosphate groups bound to a five-carbon sugar and linked to a pyrimidine or purine base, the molecules have a negative charge with a pH of >2 [20]. A mobile gradient was used consisting of the following: mobile phase A – 10 mM ammonium acetate in acetonitrile/H_2_O (30:70, v/v) (adjusted to pH 5.5) and mobile phase B – 20 mM ammonium acetate in acetonitrile/H_2_O (30:70, v/v) (adjusted pH 10.5). The flow rate was 0.25 ml/min under a gradient elution: 0–0.5 min, 10% B; 0.51–3.0 min, 50% B; 3.0–3.5 min, 50–100% B; 3.5–6.5 min 100% B; 6.6–12.0 min, 10% B over a total run time of 12 min. The autosampler was set at 10°C and the injection volume was 15 μl. The needle was washed with 500 μl wash solution before and after aspiration (water:acetonitrile:ammonium hydroxide solution [80:20:0.2]) between injections.

The MS was operated in positive ionization mode to produce characteristic fragmentation patterns for all analytes which are detailed in Supplementary Table 1, along with the mass spectrometric instrument parameters.

### Plasma separation

**Figure 1.**
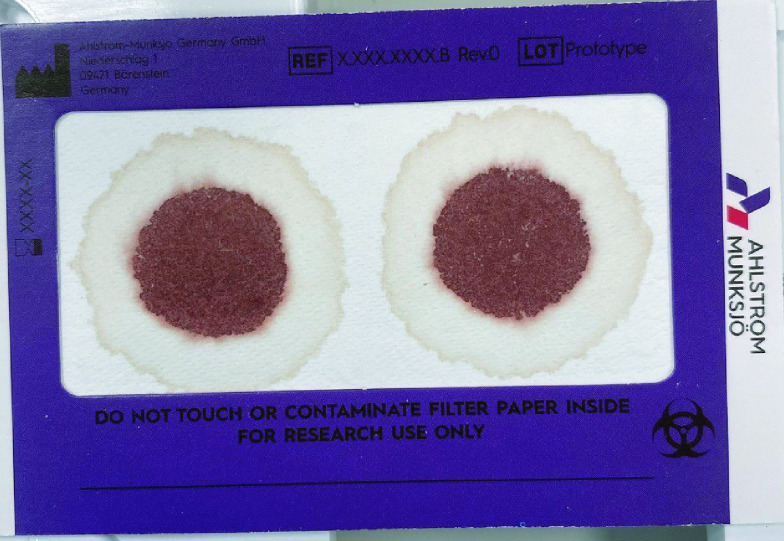
Whole blood spotted onto HemaSep card (100 μl per spot), which separates out into distinct cellular and plasma fractions upon contact.

To separate plasma using the HemaSep technology, whole blood (100 μl) was spotted onto each card in duplicate. The blood was left for 10–20 min to separate completely, followed by a drying time of 2 h. The inner fraction contains all cellular components of whole blood, which is surrounded by a distinct plasma ring (Figure 1), thus allowing for simultaneous detection of both parent and metabolite NRTI from a single whole blood sample.

### Preparation of stock solutions, standards & quality controls

A 1 mg/ml stock solution of each metabolite was prepared by dissolving 1 mg of drug (TFV-DP, FTC-TP, 3TC-TP) to MeOH (1:1 ratio of free drug:MeOH). Eight intermediate standard solutions (62.5, 125.0, 312.5, 1250.0, 3125.0, 6250.0, 10000.0, 12500.0 nM [or pmol/ml]) and four quality control (QC) intermediates at the lower limit of quantitation (LLOQ) (62.25 nM), low (LQC; 100 nM), medium (MQC; 1000 nM) and high (HQC; 10000 nM) were prepared by serial dilution of this stock to create intermediate solutions in 50:50 MeOH:H_2_O. QCs were prepared in a similar manner. Following this, 100 μl whole blood was spotted onto HemaSep cards and allowed to dry for up to 1 h, followed by the addition of 20 μl of each intermediate solution on top of individual semidry blood spots to create final concentrations of eight standards (1.25, 2.5, 6.25, 25, 62.5, 125, 200, 250 nM) and four QCs (1.25, 2, 20, 200 nM). These were left to dry overnight at ambient temperature before being stored at -80°C prior to analysis. A stable isotope labelled IS (^13^C_5_-TFV-DP in 20 μM stock) intermediate solution (2 μM) was made by diluting 20 μl of stock with 1980 μl 50:50 MeOH:H_2_O and stored at -40°C. In order to create the desired working solution (1 μg/ml), 2 μM intermediate solution was diluted 1:10 with 50:50 MeOH:H_2_O.

### Extraction procedure

The whole inner spot (~12 mm; RBC section) was punched out of the HemaSep card using a 12 mm punch for metabolite extraction. In the case of a visibly abnormal spot size, the level of variation (i.e., larger or smaller than usual) was assessed and such spots were cut out manually using scissors. Each punch was added to individual 5 ml glass tubes, and soaked in 70:30 MeOH:20% formic acid, with the addition of 20 μl of IS (1 μg/ml), followed by sonication for 30 min. Oasis weak anion exchange SPE cartridges (30 mg) were preconditioned using 1 ml MeOH and centrifuged at 1500 r.p.m. for 1 min, followed by a second conditioning using 1 ml 2:25:73 formic acid:MeOH:H_2_O and centrifuged again under the same conditions. Following this, the extracted samples were loaded and centrifuged, followed with two wash steps using 1 ml H_2_O and 1 ml 50:50 MeOH:H_2_O. Finally, the samples were eluted into fresh 5 ml tubes using 1 ml 2:25:73 (NH_3_OH:H_2_O:ACN). The eluate was left to dry overnight at ambient temperature under a constant stream of nitrogen before being reconstituted the following day in 200 μl 70:30 MeOH:H_2_O and injected on the SCIEX 5500 triple quadrupole LC–MS system.

### Method validation

This novel LC–MS method was validated in accordance with the defined FDA guidelines and acceptance criteria for bioanalytical method validation [19].

#### Selectivity

Selectivity was determined by comparing the level of interference from six individually extracted blank DBSs against the concentration at the LLOQ. Any peak area responses from noise interference at the expected retention time of the analytes were acceptable, providing that the interference was <20% of the mean response of the LLOQ (n = 6). In regards to the IS, <5% of the mean peak area response in the six LLOQ samples was acceptable.

#### Carryover

Carryover was evaluated via injection of extracted HemaSep DBS calibrators at the assay ULOQ, after which two sequential extracted blank HemaSep DBSs were injected, performed over three separate occasions. Carryover was calculated as percentages of both the LLOQ and ULOQ, neither of which should exceed >20% of the LLOQ.

#### Precision & accuracy

Precision and accuracy were assessed through the analysis of three separate assays, consisting of a double blank, blank, eight calibration standards (in duplicate) and six QCs from each level (LLOQ, LQC, MQC and HQC), run over 3 days. Acceptance criteria stipulated that any deviation from the nominal calibrator and QC concentration of each analyte must be ≤±15%, with exception of the LLOQ whereby ≤±20% is acceptable. Accuracy was characterized as the percentage deviation of the back-calculated analyte concentration from the nominal QC concentration, and precision was expressed as a percentage CV (%CV).

#### Recovery & matrix effect

The percentage recovery (process efficiency [%PE]) and matrix effects (%ME) were assessed quantitatively using methods described by Matuszewsi *et al*. [21]. A single lot of whole blood was purchased through the National Frozen Blood Bank (Speke, Liverpool, UK) and was supplied in a large volume from one healthy volunteer. There were difficulties in obtaining sufficient blood samples under our in-house biological sampling ethics due to the availability of healthy donors and restrictions on the amount of blood that could be taken. The peak area responses of pre-extracted (n = 6) HemaSep DBS QC (C), post-extracted HemaSep DBS blanks spiked at the corresponding QC concentration (B) and metabolite spiked directly into reconstitution solution (70:30 MeOH:H_2_O [v/v]) in the absence of matrix (A) were all compared. The %PE was calculated by analysing the peak area response of pre-extracted HemaSep DBS compared with the peak area response of mobile phase spiked with metabolite at the corresponding concentration (C/A*100). The %ME was deduced by analysis of the peak area response of each analyte spiked into extracted blank HemaSep DBS extracts compared with the peak areas at a comparable concentration in mobile phase (B/A*100). The IS-normalized recovery was determined by analysis of the peak area ratio of the pre-extracted (C2) compared with post-extracted (B2) HemaSep DBS QC (C2/B2*100).

#### Reinjection/reproducibility

To evaluate stability of extracted samples in the autosampler at 4°C, an accepted precision and accuracy batch was reinjected 48 h after the initially injected run. The responses between original and reinjected runs were compared with determine analyte autosampler stability.

#### Hematocrit variation

Hematocrit variation was tested by using whole blood to simulate different percentage hematocrit (20–80%), considering extremes at both the low and high end. Whole blood was collected in an EDTA tube and centrifuged at 2000 r.p.m. for 10 min to separate out the plasma and packed cells. The plasma was then removed and transferred to a fresh tube. Separate volumes of packed cells were then diluted with plasma to create 20, 40, 60 and 80% hematocrit. Blood from each hematocrit level was then spotted onto HemaSep cards (100 μl per spot), before 20 μl of aqueous LQC and HQC were spiked on top of each spot. The cards were left to dry overnight at ambient temperature before being stored at -80°C in sealed bags containing desiccant. The DBSs were extracted as previously described and read off an extracted calibration curve. The back-calculated concentrations, at each simulated % hematocrit, were compared against their respective nominal QC value.

#### Stability

##### Pre-spotting stability of clinical samples

The stability of TFV-DP and FTC-TP in whole blood and the optimum time from collection to spotting onto the cards was determined. EDTA whole blood was collected from PLWH (n = 3) receiving TDF- or TAF- containing regimens. The blood was spotted onto HemaSep cards immediately (t = 0; control) and after 5 h at room temperature following collection. A timeframe of 5 h was selected as an initial indicator of transport time in a field-related setting The cards were allowed to dry for 1 h before being put in a sealed bag containing desiccant and stored in a -80°C freezer, prior to analysis.

##### Post-spotting stability of clinical samples

In order to test benchtop stability of clinical DBS, HemaSep cards were spotted with whole blood from PLWH and left at ambient temperature for 11 days (n = 1) and 29 days (n = 3) and compared with immediately spotted samples (T = 0). Following this, the spots were analyzed using the aforementioned 70:30 MeOH:20% formic acid extraction method.

##### Long-term stability

To gain a greater insight into the potential long-term stability of the metabolites from HemaSep DBS, QCs at low, medium and high concentrations were prepared, spotted onto cards and stored for approximately 5 months at -80°C. The stability of each analyte was then evaluated by running the stored QC samples off a freshly prepared calibration curve. Samples were considered to be stable if values were found to be within the acceptance limits of accuracy (±15% of their nominal concentration) and precision (≤15%CV).

### Clinical application of the method

Whole blood was collected from 12 PLWH (receiving either TDF- or TAF-based ART regimens) attending the Royal Liverpool University Hospital, Liverpool, UK between June 2020 and February 2023. The whole blood was spotted within 60 min of collection onto HemaSep cards (100 μl per spot) and stored at -80°C. The whole inner spot (RBC fraction) was punched out of the HemaSep card and extracted as previously described.

## Results

### Method optimization

Early method development investigations included optimizing the extraction method using various ratios of MeOH:formic acid, testing the effect of whole blood spot volume on response (50 μl vs 100 μl), determining the optimal time for spotting aqueous solution onto calibrators, comparing punch size and/or location, and improving recovery and reducing matrix suppression using various SPE clean-up methods.

It was determined that an extraction of 70:30 MeOH:20% formic acid on the whole inner RBC spot (100 μl) followed by a weak anion exchange SPE clean up provided optimal recovery compared with other variations previously tested. The whole spot method was chosen over a 6 mm sub-punch to improve the sensitivity of the method, as 6 mm sub-punches did not provide a sufficient signal intensity. Punch location did not have any substantial effect on recovery, a finding that is supported by a previous study by Zheng and colleagues [12]. The metabolites were not detected in the plasma (outer ring) section of the spot in both spiked and incurred samples, thus verifying that metabolites did not diffuse into the plasma and remained within the inner RBC fraction. Therefore, the impact on the quantitative readout from partially capturing sections of the plasma (outer ring) during punching of visibly abnormal spots will be negligible.

### LC–MS conditions

**Figure 2.**
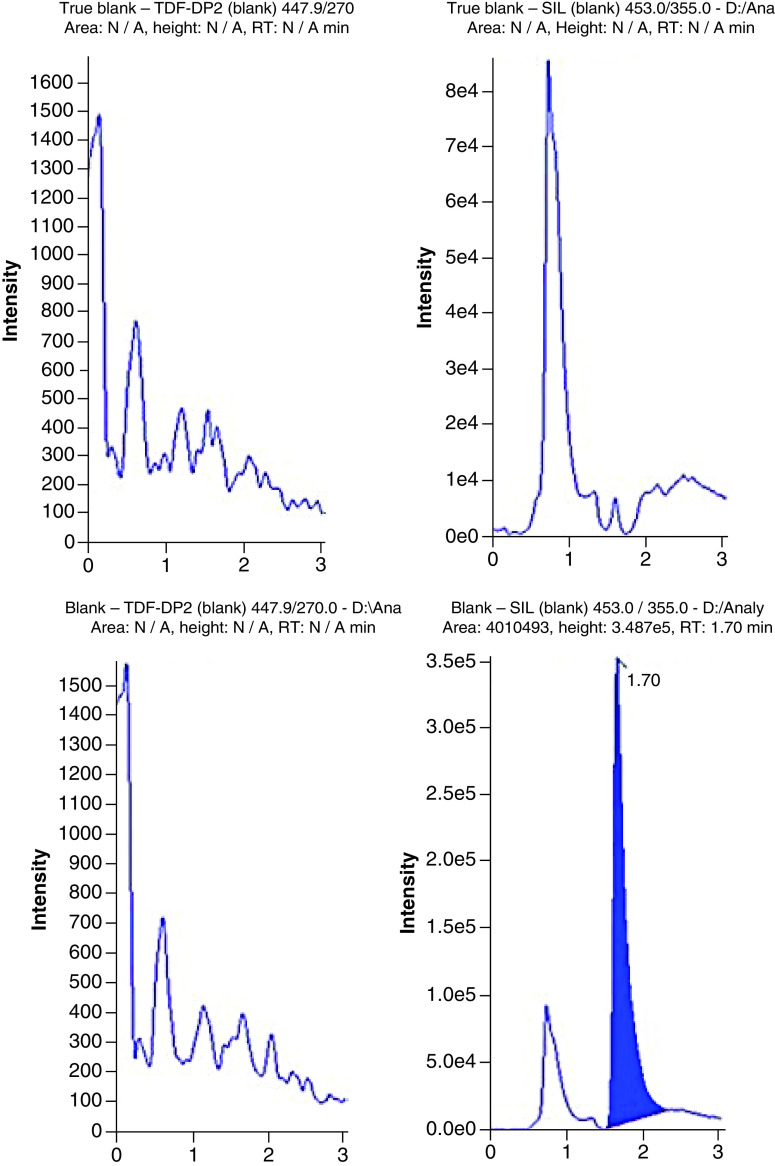

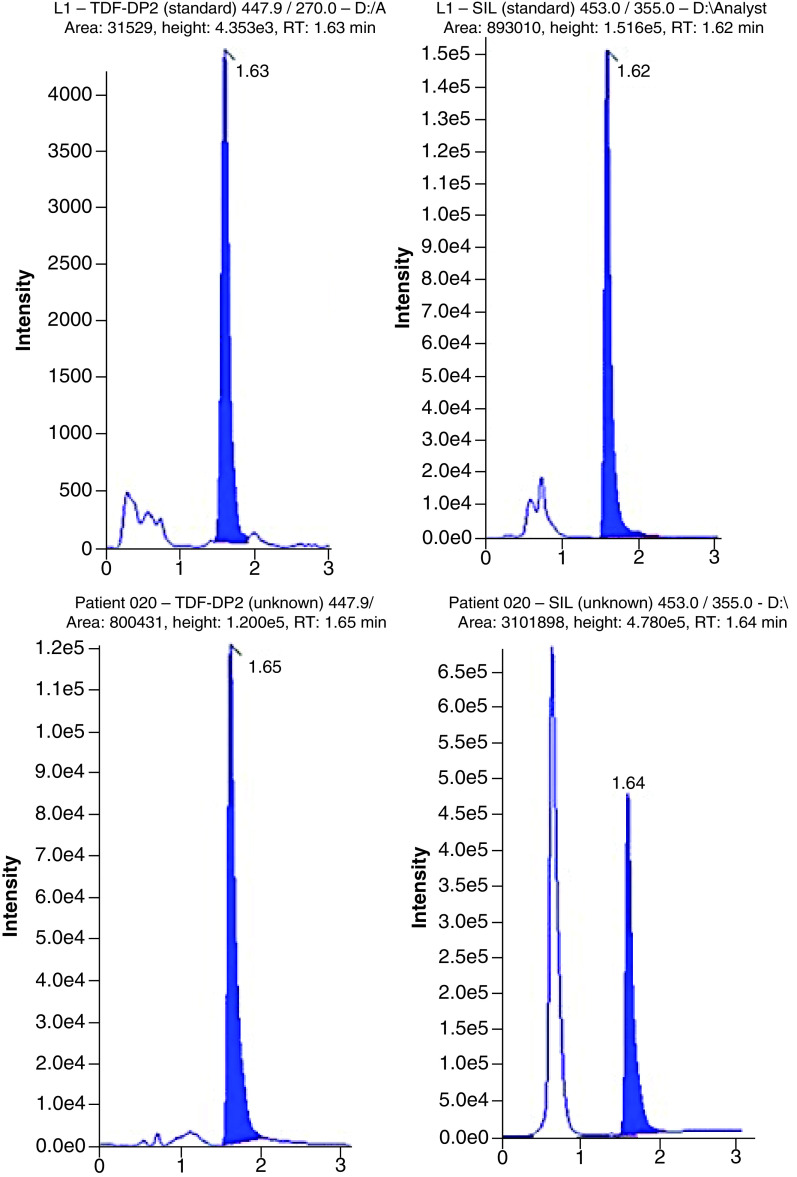
Chromatograms showing the quantitation of tenofovir diphosphate from extracted blanks (with without internal standard; true blank vs blank), at the lower limit of quantification, and from an extracted patient HemaSep dried blood spot sample (tenofovir diphosphate: 105.97 pmol/sample). Sample = whole inner red blood cell spot (∼12 mm). SIL: Stable isotopically labelled internal standard. RT: Retention time.

**Figure 3.**
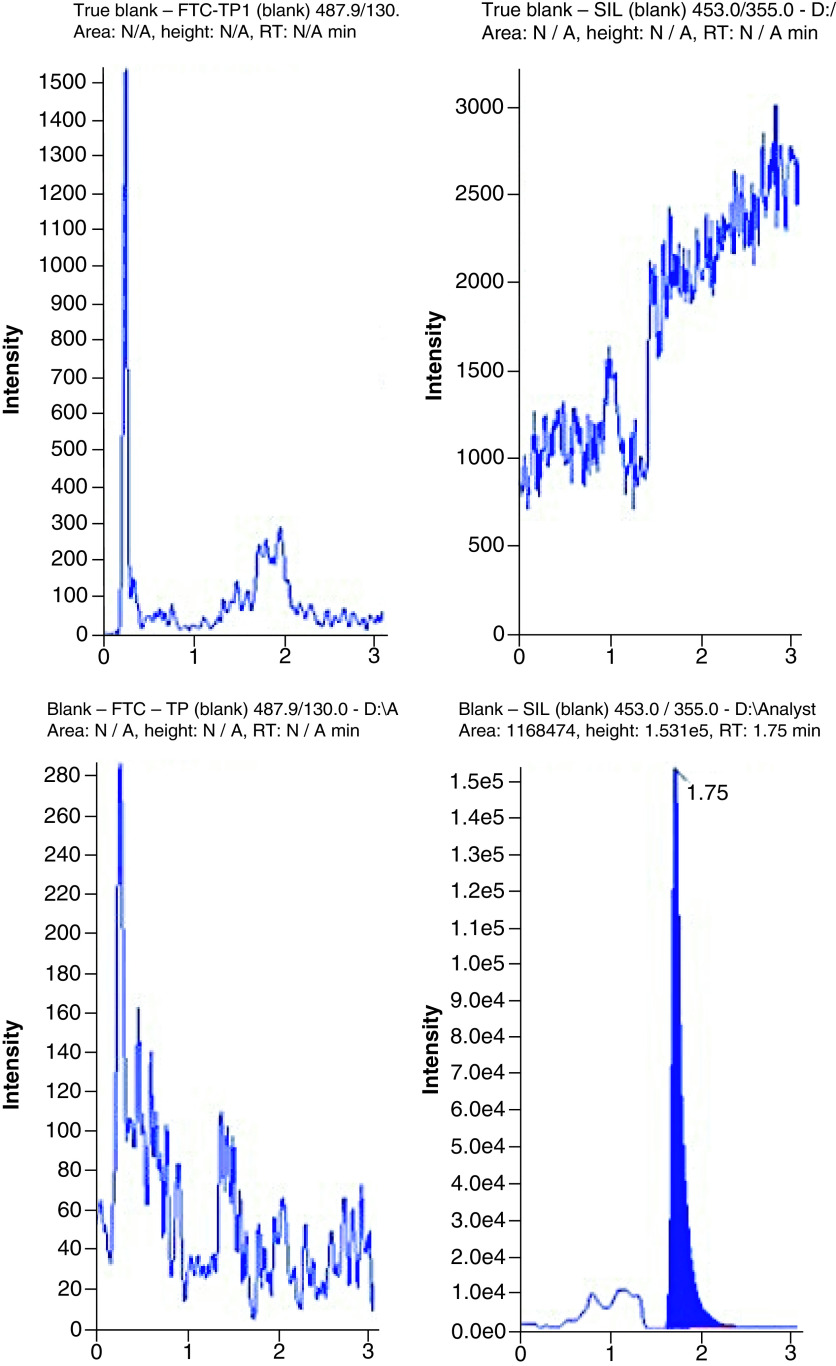

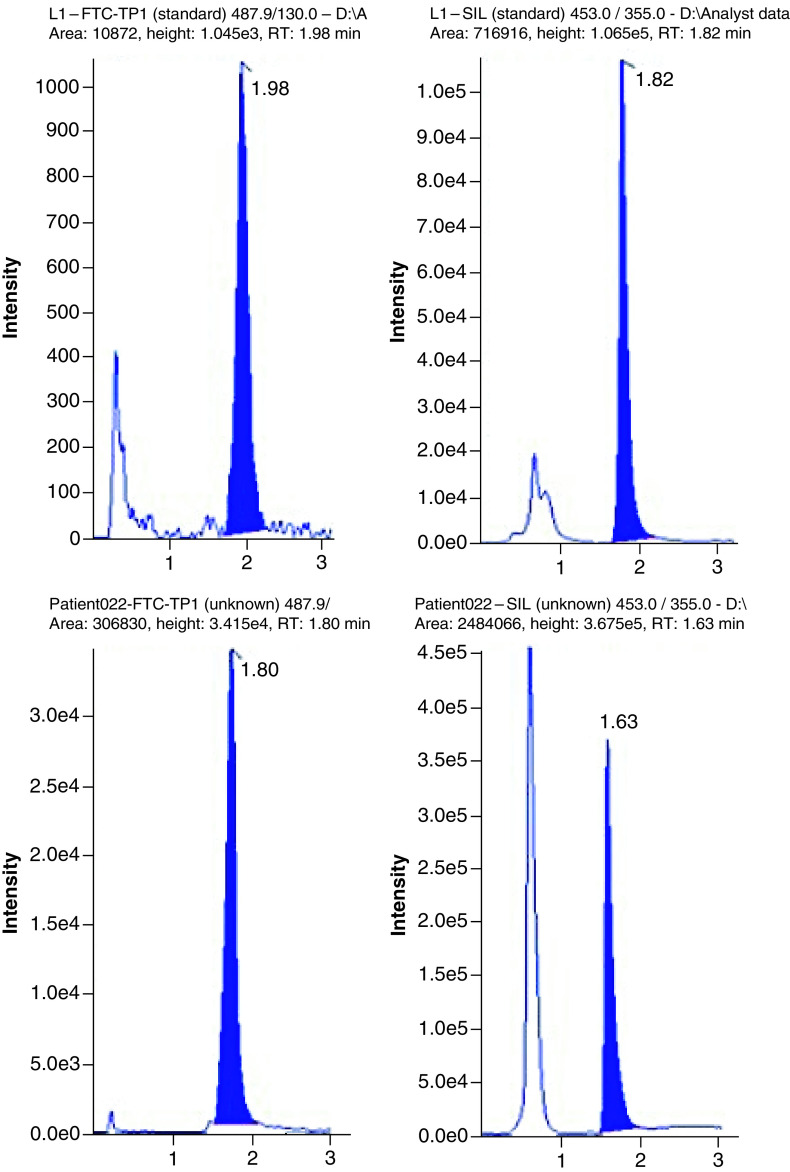
Chromatograms showing the quantitation of emtricitabine triphosphate from extracted blanks (with and without internal standard; true blank vs blank), at the lower limit of quantification, and from an extracted patient HemaSep dried blood spot sample (emtricitabine triphosphate: 27.73 pmol/sample). Sample = whole inner red blood cell spot (∼12 mm). SIL: Stable isotopically labelled internal standard. RT: Retention time.

**Figure 4.**
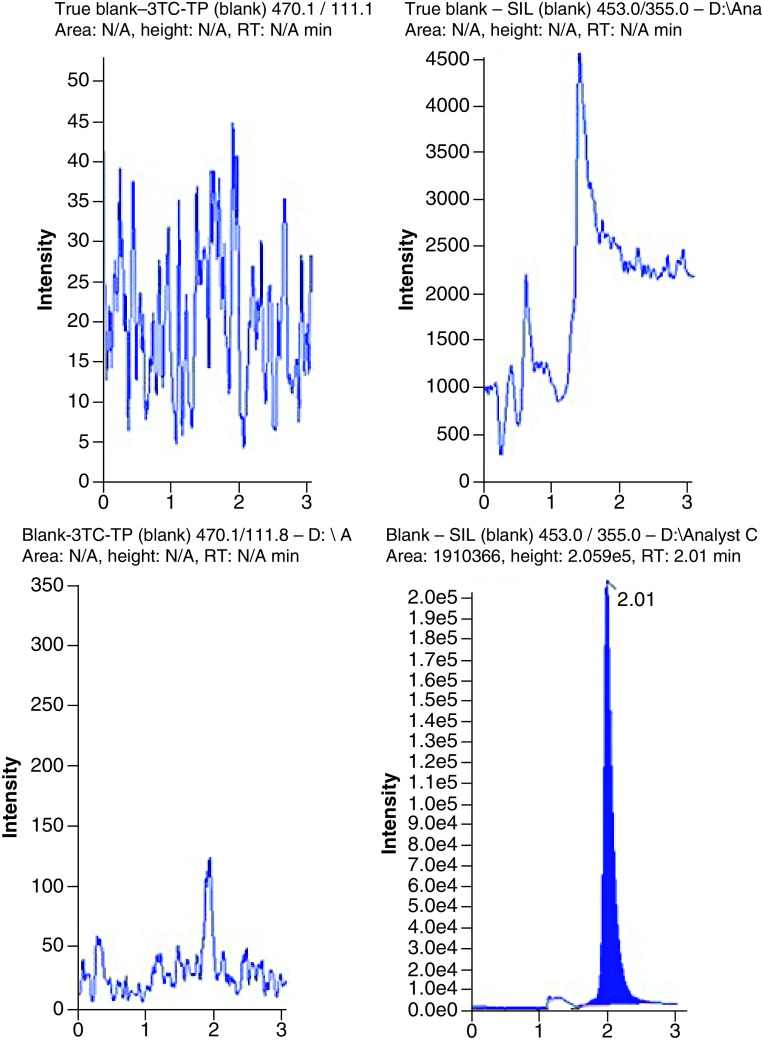

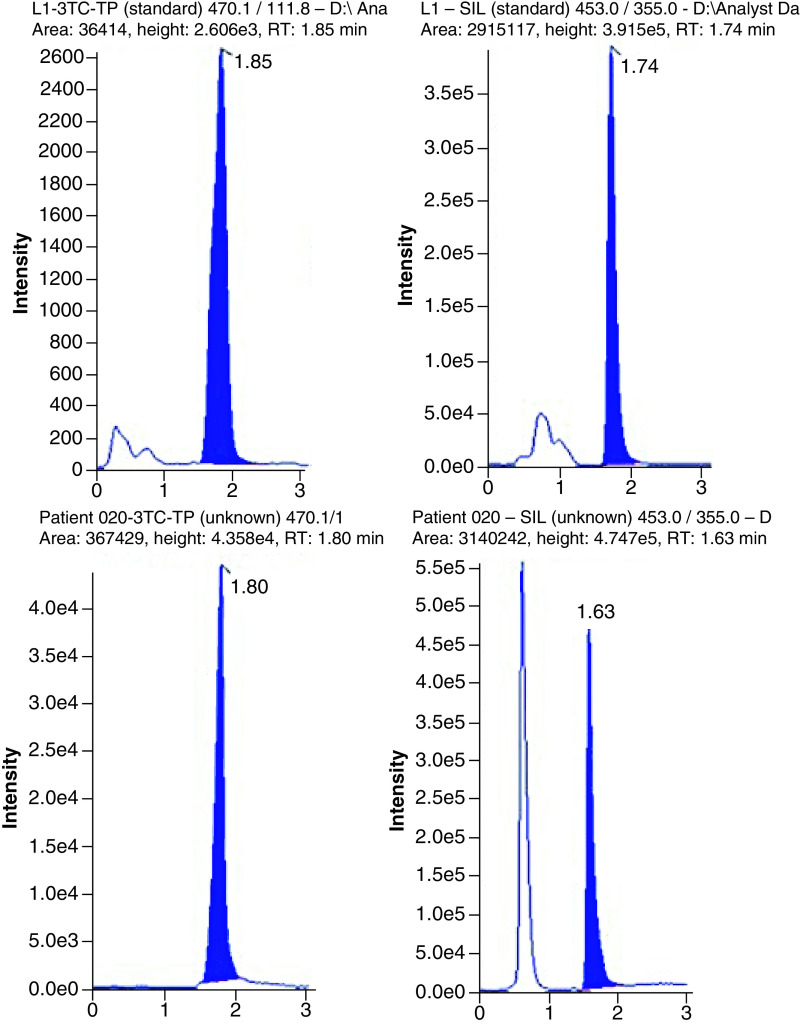
Chromatograms showing the quantitation of lamivudine triphosphate from extracted blanks (with and without internal standard; true blank vs blank), at the lower limit of quantification, and from an extracted patient HemaSep dried blood spot sample (lamivudine triphosphate: 66.53 pmol/sample). Sample = whole inner red blood cell spot (∼12 mm). SIL: Stable isotopically labelled internal standard. RT: Retention time.

An anion exchange column (Thermo Fisher BioBasic™ AX 50 × 1 mm) and a pH gradient was used to chromatographically resolve the phosphorylated metabolites. Representative chromatograms are shown in Figures 2, 3 & 4 for TFV-DP, FTC-TP and 3TC-TP, respectively. The analytes eluted from the column at 1.59 min for TFV-DP, 1.63 min for FTC-TP and 1.49 min for 3TC-TP. The IS (^13^C_5_-TFV-DP) eluted at 1.57 min.

### Method validation

#### Selectivity

The background interference from blank DBS extracts (n = 6) at the retention time of all analytes was found to be <20% of the signal intensity of the LLOQ of the assay. In addition, the background noise in the extracted blank HemaSep DBS was negligible for the IS (^*1*3^C_5_-TFV-DP) for all batches tested.

#### Carryover

Carryover into a blank extract following the injection of a ULOQ extract (250 pmol/sample) was <20% of the LLOQ and ULOQ for all analytes. In addition, there was no area response from IS in the blank samples following injection of the ULOQ.

#### Precision & accuracy

The precision and accuracy for TFV-DP, FTC-TP and 3TC-TP are shown in Table 1. The calibration range was linear with a weighing factor of 1/x^2^ from 1.25 pmol/sample to 250 pmol/sample. The mean regression coefficients (r^2^) were 0.998, 0.997 and 0.998 for TFV-DP, FTC-TP and 3TC-TP, respectively. All standard and QC levels were within ±15% (and ±20% at the LLOQ).

**Table 1.** Precision and accuracy for tenofovir diphosphate, emtricitabine triphosphate and lamivudine triphosphate in HemaSep dried blood spot (red blood cell fraction) in quality control samples based on three separate runs.

	Tenofovir diphosphate				Emtricitabine triphosphate				Lamivudine triphosphate			
	LLOQ	LQC	MQC	HQC	LLOQ	LQC	MQC	HQC	LLOQ	LQC	MQC	HQC
Nominal-quality control (pmol/sample)	1.25	2	20	200	1.25	2	20	200	1.25	2	20	200
Mean value (pmol/sample)	1.37	2.05	20.98	214.33	1.26	2.11	22.26	221.52	1.30	2.10	21.20	209.47
%CV (interday)	7.42	8.62	10.53	8.00	9.21	10.51	10.90	10.89	10.73	11.91	10.81	8.54
%Accuracy (interday)	9.47	2.70	4.87	7.17	0.62	8.35	11.30	10.76	4.18	4.75	5.98	4.73
%CV (intraday)	5.39	8.64	8.17	5.89	6.52	6.87	9.31	7.25	6.04	7.60	10.15	6.49
%Accuracy (intraday)	10.93	4.29	7.10	7.23	5.73	12.13	13.00	11.87	9.20	10.3	8.72	3.30

n = 6 in one run (intra); n = 6 in three individual runs (inter). Acceptability criteria for range of accuracy is ±15% (±20% for LLOQ).

HQC: High-quality control; LLOQ: Lower limit of quantification; LQC: Low-quality control; MQC: Medium-quality control.

#### Recovery & matrix effect

The mean (±CV %), percentage recovery, %PE and %ME of TFV-DP, FTC-TP and 3TC-TP are shown in Table 2. Recovery from HemaSep DBS was >93% for all analytes tested and was consistent at low, medium and high concentrations over the calibration range (%CV <15%). Given the high recovery for each analyte, it is unlikely that any significant matrix effect is present for this method.

**Table 2.** Recovery & matrix effect of tenofovir diphosphate, emtricitabine triphosphate and lamivudine triphosphate in HemaSep dried blood spot (red blood cell fraction).

	Tenofovir diphosphate				Emtricitabine triphosphate				Lamivudine triphosphate			
	LQC	MQC	HQC	%CV	LQC	MQC	HQC	%CV	LQC	MQC	HQC	%CV
Recovery % (% process efficiency)	98.24	93.52	98.97	3.10	108.08	110.70	116.48	3.85	125.12	108.86	112.97	7.31
Matrix effect, %	94.89	98.40	99.10	2.30	94.35	98.96	100.34	3.20	100.55	99.62	100.74	0.60
Analysis recovery %	71.63	62.70	70.78	7.21	79.27	74.20	82.75	5.46	87.04	72.94	80.02	8.81

n = 6.

HQC: High-quality control; LQC: Low-quality control; MQC: Medium-quality control.

#### Reinjection/Reproducibility

All standard and QC levels were within ±15% (and ±20% at the LLOQ) following reinjection of an accepted batch after the samples had been stored in the instrument autosampler (4°C) for 48 h.

#### Hematocrit variation

Results from the hematocrit investigation can be found in Supplementary Figure 1. Metabolite concentrations obtained from HemaSep cards spotted with a simulated hematocrit of 40% were within 15% of their nominal QC value and back-calculated concentrations remained within 15% for hematocrits of 20–50% (TFV-DP) and 40–50% (FTC-TP/3TC-TP), respectively. However, for extremes in hematocrit, the back-calculated concentrations exceeded the 15% cut-off – absolute concentrations increased with low (<20%) hematocrits and were suppressed at higher (>50%) hematocrits, as depicted in Supplementary Figure 1.

#### Stability

Full data for stability testing can be found in Table 3A & B. The stability of 3TC-TP in whole blood, pre- and post-spotting, was not evaluated using incurred samples due to a limited number of patients receiving this formulation at the time of experimentation.

**Table 3.** Long- and short-term stability testing results for tenofovir diphosphate, emtricitabine triphosphate and lamivudine triphosphate in HemaSep dried blood spot (red blood cell fraction) (expressed as percentage difference from control).

(A)			
	Tenofovir diphosphate	Emtricitabine triphosphate	Lamivudine triphosphate
Long-term stability (5 months, -80°C) (n = 6)			
Mean stability	113.9%	85.1%	101.9%
%CV	11.2%	14.4%	13.8%
Reinjection reproducibility (48 hr, 4°C) (n = 6)			
Mean bias (%)	2.0%	6.7%	1.2%
%CV	7.2%	6.2%	5.8%

**(B)**			
**Patient sample stability**			
**Benchtop (5 h pre-spotting, ambient temperature) (2 replicates/sample)**	**Tenofovir diphosphate**	**Emtricitabine triphosphate**	**Lamivudine triphosphate**
Mean stability	118.2%	131.9%	NA
%CV	16.9%	16.8%	
Short-term stability (29 days, ambient temperature) (2 replicates/sample)			
Mean stability	83.6%	84.0%	NA
%CV	10.1%	8.7%	
Short-term stability (11 days, ambient temperature) (2 replicates/sample)			
Mean stability	88.7%	NA	NA
%CV	8.3%		

Long-term stability testing and reinjection reproducibility (Table 3A) was performed using HemaSep dried blood spot calibrators prepared with blank whole blood spiked with known concentrations of aqueous drug (n = 6). Short-term stability testing (Table 3B) was carried out using three patient samples (run in duplicate) obtained from the Royal Liverpool University Hospital.

NA: Not applicable.

##### Pre-spotting stability of clinical samples

Extraction was performed on the HIV+ patient whole blood that had been spotted immediately (t = 0; control) or after 5 hrs of storage on the benchtop. When analyzing the percentage deviation from control, an enhancement in the area response was observed when the whole blood had been left out on the bench for 5 h, with TFV-DP increasing by 18% and FTC-TP by 31% in relation to blood that had been immediately processed (t = 0).

##### Post-spotting stability of clinical samples

After storage of the HemaSep DBS for 29 days at ambient temperature, the percentage difference from control for the benchtop DBS decreased by approximately 16% for both TFV-DP and FTC-TP, as compared with immediately spotted DBS stored at -80°C. Stability of TFV-DP was also assessed after storage of HemaSep DBS for 11 days at ambient temperature. The stability decreased by approximately 11% compared with immediately spotted DBS stored at -80°C.

##### Long-term stability

After being stored at -80°C for 5 months, HemaSep DBS low, medium and high concentration QCs (n = 6 from each level) were run alongside a freshly prepared calibration curve and QCs. TFV-DP, FTC-TP and 3TC-TP were found to maintain stability after 5 months' storage at -80°C, within the acceptable limits of accuracy (±15%).

### Clinical application of the method

Metabolite concentrations in patients receiving ART are shown in Table 4. Of those receiving TAF 10 mg (n = 2), only one patient had detectable levels of TFV-DP (3.06 pmol/sample) and FTC-TP (3.21 pmol/sample). Geometric mean (95% CI) TFV-DP concentrations were 2.81 pmol/sample (1.60–4.03) in patients (n = 7) receiving TAF (25 mg) regimens and 42.05 pmol/sample (23.84–60.26) for those (n = 3) on TDF (245 mg). FTC-TP geometric mean (95% CI) values were 3.32 pmol/sample (2.34–4.30) for patients on TAF (25 mg) and 3.86 pmol/sample (0.88–6.84) for those taking TDF (245 mg). A single patient receiving 3TC had a 3TC-TP concentration of 16.08 pmol/sample.

**Table 4.** Amounts of tenofovir diphosphate, emtricitabine triphosphate and lamivudine triphosphate extracted from HemaSep dried blood spot (red blood cell fraction) from patients receiving emtricitabine/tenofovir alafenamide- or tenofovir disoproxil fumarate-based antiretroviral therapy regimens.

TAF/TDF regimen	Fixed-dose combination	Time post-dose (h)	Tenofovir diphosphate (pmol/sample)	Emtricitabine triphosphate (pmol/sample)	Lamivudine triphosphate (pmol/sample)
TAF	TAF 10 mg/FTC 200 mg	12	3.06	3.21	<LLQ
TAF	TAF 10 mg/FTC 200 mg	14	<LLQ	<LLQ	<LLQ
**Geometric mean (95% CI)**		12.96 (11–14.92)	NA	NA	NA
TAF	TAF 25 mg/FTC 200 mg	3.7	2.43	3.26	<LLQ
TAF	TAF 25 mg/FTC 200 mg	24	<LLQ	2.91	<LLQ
TAF	TAF 25 mg/FTC 200 mg	13	3.78	5.32	<LLQ
TAF	TAF 25 mg/FTC 200 mg	14	3.38	3.37	<LLQ
TAF	TAF 25 mg/FTC 200 mg	4.5	5.64	2.38	<LLQ
TAF	TAF 25 mg/FTC 200 mg	13	1.37	<LLQ	<LLQ
TAF	TAF 25 mg/FTC 200 mg	5	2.06	<LLQ	<LLQ
**Geometric mean (95% CI)**		8.98 (3.60–14.37)	2.81 (1.60–4.03)	3.32 (2.34–4.30)	NA
TDF	TDF 245 mg/FTC 200 mg	12	45.2	5.67	<LLQ
TDF	TDF 245 mg/3TC 300 mg	4	27.56	<LLQ	16.08
TDF	TDF 245 mg/FTC 200 mg	15	59.69	2.63	<LLQ
**Geometric mean (95% CI)**		8.96 (2.53–15.40)	42.05 (23.84–60.26)	3.86 (0.88–6.84)	NA

Sample = whole inner red blood cell spot (∼12 mm).

FTC: Emtricitabine; <LLQ: Less than lower limit of quantitation; NA: not applicable; TAF: Tenofovir alafenamide; TDF: Tenofovir disoproxil fumarate.

## Discussion

Here we describe the development and application of a novel method for the extraction and quantification of NRTI metabolites from plasma separation cards (HemaSep DBS) using LC–MS. The analytical method achieved high recovery of all phosphorylated metabolites with minimal matrix effect and carryover. The cards themselves allow for the stability of NRTI metabolites to be maintained in both the short and long term, including minimal (16%) reduction in stability when stored at ambient temperature for at least 29 days.

HemaSep DBSs are a novel and promising method of microsampling that enable the rapid separation of plasma from whole blood at site, which removes the requirement of a centrifuge making them ideal for use in clinics in resource-limited settings. However, due to the lateral flow technology, plasma separation cards, such HemaSep DBS, may be more susceptible to variable volumes of blood, which is a common occurrence in remote clinics where pipettes are not routinely available or calibrated. Testing is therefore ongoing using real-life clinical samples (such as fingerstick sampling) to determine how different blood volumes affect the whole blood partitioning process, spot size and the quantitative readout.

A significant obstacle of DBS is the potential for sampling bias due to uncontrolled saturation or inconsistent spreading of the liquid blood as a result of the variation in hematocrit, which can, in turn, affect the quantitative output or reproducibility of the data [22]. Our experimental results showed that the quantitative values were perturbed with simulated hematocrits of less than 20% and greater than 50% (Supplementary Figure 1) which implies that the HemaSep cards have a relatively narrow operating window and do not sufficiently correct for extremes in hematocrit. These preliminary data therefore suggest that HemaSep may only be suitable for whole blood specimens that fall within a normal hematocrit range of 30–50% for healthy adults [23]. However, given the nature of the experimental conditions, that is, spiking of working QC directly onto the spot, these data should be interpreted with caution and may not fully represent the effect of variations in hematocrit seen in ‘real-life’ clinical samples. Application of aqueous QC solution (20 μl) onto the surface of the spot captures the extracellular dispersion of the QC and does not truly mimic the behavior of the phosphorylated metabolites confined in the cells. Furthermore, the quantitative readouts at the extremes of hematocrit (20 and 80%) were more susceptible to methodological factors, such as the degree of matrix suppression and amount of metabolite extracted from the spot as compared with the reference calibrators which were spotted using normal whole blood (∼45% hematocrit). Moreover, the stable isotope-labeled IS (^13^C_5_-TFV-DP) did not fully compensate for the matrix-related effects or variations in the extraction yield, as this was added to the surrounding extraction solution, as opposed to being applied directly to the surface to the spot; in addition, it was not possible to commercially source stable isotopically labelled IS for FTC-TP and 3TC-TP. Variation seen in concentration may also be mitigated by performing a 6 mm subpunch extraction, as inner spot size may vary depending on the percentage hematocrit spotted onto the card. Further experiments are therefore warranted investigating the effect of hematocrit in whole blood specimens taken from PLHW receiving NRTI-based therapies.

Stability testing revealed that in the short term, when whole blood is left at ambient temperature for 5 h prior to spotting, an enhancement effect is seen. This was particularly pronounced for FTC-TP, in that concentrations increased by ∼30% when compared with immediately spotted samples. These observations are consistent with previous reports of TFV-DP and FTC-TP stability in whole blood. Zheng and colleagues found that increased FTC in plasma was correlated with increased FTC-TP in RBC when whole blood is left at room temperature for 6–24 h (compared with immediately spotted, 6 h: 21.7% increase; 24 h: 49.9% increase) [12]. Our data suggests that even when left for shorter periods of time at ambient temperature before spotting, concentrations may not reflect that at the time of sampling due to the ongoing accumulation of FTC-TP in RBC. Future work is ongoing to ascertain a more precise stability window between 1–4 h. However, based on these findings, it is recommended that blood is spotted as soon as possible and within an hour of collection.

There was evidence of TFV-DP and FTC-TP degradation (a decrease of ∼16% relative to control) when HemaSep cards were kept at ambient temperatures for up to 29 days. Previous studies, with Whatman 903 Protein Saver cards, have reported that nucleoside metabolites remain stable at ambient temperatures for up to 3 days (3TC-TP), 5 days (TFV-DP) and 8 days (FTC-TP), respectively [12,13]. Further work is ongoing to determine analyte stability when stored at ambient temperature for shorter time periods, such as 7, 14 and 21 days, in order to identify a viable stability window. The stability of 3TC-TP in whole blood, both pre- and post-spotting, was not evaluated using clinical samples due to a limited number of patients receiving 3TC-based regimens at the time of experimentation, which is a consideration for future work.

Long-term stability testing revealed that TFV-DP, FTC-TP and 3TC-TP maintain stability on HemaSep DBS for up to 5 months when stored at -80°C. The percentage stability for FTC-TP was borderline (85.1%), suggesting further stability tests for FTC-TP are required over a shorter storage duration (2–3 months). The stability data were presented as averages on the grounds that select outliers resulted in stability falling outside of the 15% acceptance criteria. Upon closer inspection of the data, we noted that there were fluctuations in the area response of the freshly prepared calibrators which we believe to be the cause of these outliers. We deemed this to be an isolated event and thus presented the data as an average as a more meaningful indication of overall stability. Given that long-term stability was only tested using ‘extracellularly’ spiked QC samples, further investigations are warranted to establish the long-term stability of the phosphorylated metabolites *in vivo* using clinical specimens obtained from patients receiving NRTI.

Nucleoside di/triphosphate concentrations were determined in sample of patients stable on ART attending the Royal Liverpool University Hospital (Table 4). The amount of phosphorylated metabolite was expressed as pmol/sample, with the ‘sample’ representative of the entire inner RBC spot (Figure 1), which equates to punch diameter of approximately 12 mm. Previously reported nucleoside di/triphosphate concentrations are based on extraction of sub-punches (3–7 mm) taken from 25 μl of whole blood spotted on Whatman 903 Protein Saver cards [8,9,12,14,15,24] with TFV-DP concentrations (TAF/TDF dosing) ranging between <0.0087 and 3.78 pmol/sample [4,8,12–15,24,25], FTC-TP concentrations from <0.0001 to 0.60 pmol/sample [13,25], and 3TC-TP concentrations between 0.1 and 1.00 pmol/sample [13].

The amount of TFV-DP recovered from the HemaSep inner spot (∼12 mm) was more than 11-fold higher than values reported using Whatman sampling in subjects receiving TDF-based treatment. Similarly, FTC-TP and 3TC-TP levels per sample were greater than sixfold and 16-fold higher compared with values reported with Whatman. However, caution should be exercised when making comparisons between the two sampling approaches. The increased amount of metabolite recovered using a HemaSep punch, as reported here, is likely attributed to a number of factors, including a higher volume of blood applied to the spot (100 μl per spot for HemaSep vs 25 μl per spot for Whatman) and a larger extracted punch diameter (12 mm vs 3 mm), which in turn equates to a greater number of cells per sample. When applying a previously calculated figure of 12 million RBC per 3 mm punch [9], to HemaSep, assuming there are 5 million RBC per 1 μl blood, we estimate that the number of RBC are likely to be in the region of 500 million per 12 mm punch. Furthermore, there will be a higher density of cells in the HemaSep RBC fraction per unit area due to the displacement of plasma, but this effect is mitigated if the whole spot is extracted.

Gradients of adherence have been established using Whatman DBS and have been shown to be a significant predictor of both ART viral suppression [24] and the effectiveness of PrEP [26]. Adherence modeling studies suggest that a concentration of TFV-DP in DBS (Whatman) for 100% adherence to daily ART at steady-state is equivalent to approximately 1560 fmol/punch, with ≥700 fmol/punch representing four or more tablets per week, 350–699 fmol/punch representing two to three tablets per week, below the <LLOQ to 349 fmol/punch indicating less than two tablets per week and <LLOQ being indicative of no doses. Given the higher di/triphosphate concentrations reported here, our data emphasize the need to determine markers of adherence using plasma separation technologies.

Interestingly, TFV-DP concentrations in the patients receiving TAF were approximately 15-fold lower compared with those that received TDF. The TAF prodrug is known to exhibit greater stability in plasma resulting in 90% lower circulating levels of parent TFV and up to sevenfold higher concentrations of the intracellular active phosphorylated moiety (TFV-DP) in PBMC [27,28]. Thus, one explanation for our finding is that lower circulating levels of parent TFV may, in turn, equate to lower levels of the active diphosphate in the RBC compartment. In addition, sites of enzymatic activity may be able to explain these differences in metabolite concentration between the two formulations. Lysosomal carboxypeptidase A (cathepsin A) is an enzyme isolated within PBMCs and has been identified as the primary hydrolase involved in the intracellular activation of TAF. As TAF is a prodrug, it requires hydrolysis by cathepsin A into the parent form TFV, which is subsequently phosphorylated into TFV-DP by intracellular kinases, which results in an increase of TFV-DP within PBMCs [29]. However, as cathepsin A is not present in RBCs, intracellular TAF is not hydrolyzed into TFV by cathepsin A, which results in lower concentrations of TFV and thus, TFV-DP, in RBCs. In the case of TDF, there is no requirement for cathepsin A activity, as it is directly phosphorylated in the cell by nucleoside diphosphate kinase and AMP kinase into TFV-DP [30], resulting in increased TFV-DP concentrations in RBCs in comparison.

These initial data for TAF are consistent with that of a previous study using Whatman DBS, whereby sub-punches had to be pooled (i.e., use of 2 × 7 mm sub-punches) in order to achieve quantifiable TFV-DP [4]. By applying an increased blood volume and taking the entirety of the RBC fraction, we were able to achieve sufficient sensitivity to quantify TFV-DP from PLWH stable on TAF using HemaSep cards. Consequently, HemaSep and other plasma separation technologies may be useful when NRTI metabolite concentrations are expected to be low, such as single-dose PK studies or in individuals taking on-demand PrEP (one or two doses before/after sex). The method is currently being applied to characterize TFV-DP (both TDF and TAF regimens) as part of an open-label drug intake cessation study in healthy volunteers (NCT04302896), where levels of the phosphorylated metabolites are anticipated to be lower.

One of the most attractive qualities of HemaSep card technology is the ability to simultaneously quantify both NRTI parent drug and metabolite from the same patient sample on the same card, as a marker of both short- and long-term adherence to NRTI treatment. We are in the process of developing a method to measure NRTI parent drug concentrations in the outer plasma ring of HemaSep cards. In addition, there is also the potential for paired drug and viral load testing, which would provide an even more comprehensive window into the success of a patient's ART regimen. TFV-DP levels in DBS are not only a strong indicator of viral suppression, but also a predictor of future onset of viremia, even in cases where the patient is currently virally suppressed [24,31].

## Conclusion

An LC–MS method has been optimized and validated for quantification of TFV-DP, FTC-TP and 3TC-TP from a novel type of plasma separation filter paper (HemaSep DBS) which allows for the separation of RBC and plasma fractions from a 100 μl spot of whole blood. These have shown to be a useful microsampling tool, which provide an easy and efficient alternative to obtaining plasma through centrifugation, while maintaining analyte stability within the matrix post-spotting. This method has been formally validated as per the FDA guidelines. This methodology and novel blood collection paper have been applied as part of a pharmacokinetic clinical trial to simultaneously quantify antiretrovirals and their respective metabolites in both plasma and cellular fractions. This will aid in providing a more realistic view of adherence in both the long term (inner RBC fraction) and short term (outer plasma ring) from a larger participant cohort. The cards will also be applied as part of clinical trials for COVID-19 drugs. Further work is ongoing to understand the effects of blood spot volume, establishing optimal timeframes to maintain analyte stability after multiple days at ambient temperature and the effects of hematocrit in real-life patient samples.

**Summary points**

- Adherence is a major determining factor in successful antiretroviral treatment and is a key area of interest in human immunodeficiency virus pharmacokinetic studies.
- Dried blood spots (DBSs) are a cheap and easy alternative to liquid plasma for pharmacokinetic studies, particularly in resource-limited settings, as they maintain stability over time.
- A novel DBS card (HemaSep) separates plasma from whole blood upon contact, eradicating need for sample centrifugation.
- HemaSep can be used to simultaneously quantify parent nucleoside/nucleotide reverse transcriptase inhibitor in plasma (outer ring of the spot), as well as their phosphorylated metabolites in the red blood cell fraction (inner spot).
- We have developed and validated a novel method for the quantification of intracellular nucleoside/nucleotide reverse transcriptase inhibitor di/triphosphates from HemaSep DBS in accordance with the US FDA guidelines for bioanalytical method validation.
- Whole blood (100 μl) was spotted onto HemaSep cards and the whole inner spot was excised, soaked in 70:30 MeOH: 20% formic acid, followed by weak anion exchange SPE and injected on a SCIEX 5500 triple quadrupole LC–MS system.
- The method was both precise and accurate over a calibration range of 1.25–250 pmol/sample with a recovery of >93% for all analytes.
- Tenofovir diphosphate maintained stability in whole blood once spotted onto HemaSep cards for up to 11 days at ambient temperature, making them cheap and easy to ship.
- Analysis of clinical samples resulted in geometric mean tenofovir diphosphate concentrations of 2.81 pmol/sample in patients (n = 7) receiving tenofovir alafenamide (25 mg) regimens and 42.05 pmol/sample for those (n = 3) on tenofovir disoproxil fumarate (245 mg).
- HemaSep has shown to be a useful microsampling tool, providing an easy and efficient alternative to liquid plasma, and maintains analyte stability at ambient temperature over time.
