# Supplementary material for: Application of novel plasma separation filter cards for quantification of nucleoside/nucleotide reverse transcriptase inhibitor di/triphosphates in dried blood spots using LC–MS
Source: Bioanalysis. 2023 Jun 9;15(13):739–56. doi: 10.4155/bio-2023-0057 (PMC10463213; doi:10.4155/bio-2023-0057)
Supplement: Supplementary file 1 [file bio-15-739-s1.docx]

**Supplementary Table**

**Supplementary Table 1** – summary of chromatographic and mass spectrometric (MS) conditions for TFV-DP, FTC-TP, 3TC-TP and ^13^C-TDV-DP internal standard (gases use arbitrary units).

Abbreviations: TFV-DP – tenofovir diphosphate; FTC-TP – emtricitabine triphosphate; 3TC-TP – lamivudine triphosphate; MS – mass spectrometric

|  | **TFV-DP** | **FTC-TP** | **3TC-TP** | **^13^C-TDF-DP** | **^MS Instrument parameters^** |  |
| --- | --- | --- | --- | --- | --- | --- |
| **Parent ion** (m/z) | 447.9 | 487.9 | 470.1 | 453.0 | Curtain gas | 30 |
| **Daughter ion** (m/z) | 270.0 | 130.0 | 111.8 | 355.0 | Collision gas | 8 |
| **Declustering potential** (V) | 60 | 40 | 90 | 90 | Ion spray voltage | 5500 |
| **Entrance potential** (V) | 10 | 10 | 10 | 10 | Temperature (^o^C) | 650 |
| **Collision energy** (V) | 38 | 25 | 25 | 25 | Ion source gas 1 | 50 |
| **Cell exit potential** (V) | 18 | 10 | 13 | 13 | Ion source gas 2 | 50 |
